# The seroprevalence of untreated chronic hepatitis C virus (HCV) infection and associated risk factors in male Irish prisoners: a cross-sectional study, 2017

**DOI:** 10.2807/1560-7917.ES.2019.24.14.1800369

**Published:** 2019-04-04

**Authors:** Desmond Crowley, John S Lambert, Graham Betts-Symonds, Walter Cullen, Mary Keevans, Enda Kelly, Eamon Laird, Tina McHugh, Susan McKiernan, Sarah Jayne Miggin, Carol Murphy, Ross Murtagh, Deirdre O'Reilly, Ciara Tobin, Marie Claire Van Hout

**Affiliations:** 1 Irish College of General Practitioners, Dublin, Ireland; 2 School of Medicine, University College Dublin, Dublin, Ireland; 3 Department of Infectious Diseases, Mater Misericordiae University Hospital, Dublin, Ireland; 4 Irish Red Cross, Dublin, Ireland; 5 Irish Prison Service, Dublin, Ireland; 6 Trinity College Dublin, Dublin, Ireland; 7 Mater Misericordiae University Hospital, Dublin, Ireland; 8 St. James' Hospital, Dublin, Ireland; 9 University of Florida, Gainesville, United States; 10 Liverpool John Moores University, Liverpool, United Kingdom

**Keywords:** hepatitis C, hepatitis C virus, injecting HCV, prisoners, prevalence, risks, blood-borne infections, epidemiology, drug users, Ireland, public health policy, viral infections

## Abstract

**Introduction:**

Data on chronic hepatitis C (HCV) infection prevalence in European prisons are incomplete and impact the public health opportunity that incarceration provides.

**Aims:**

We aimed to estimate the seroprevalence of untreated chronic HCV infection and to identify associated risk factors in an Irish male prison.

**Methods:**

We conducted a cross-sectional study involving a researcher-administered questionnaire, review of medical records and HCV serology.

**Results:**

Of 422 prisoners (78.0% of the study population) who participated in the study, 298 (70.6%) completed the questionnaire and 403 (95.5%) were tested for HCV antibodies. Of those tested, 92 (22.8%) were HCV antibody-positive, and of those, 53 (57.6%) were HCV RNA-positive, 23 (25.0%) had spontaneous clearance, 16 (17.4%) had a sustained viral response, 10 (11.0%) were co-infected with HIV and six (6.0%) with HBV. The untreated chronic HCV seroprevalence estimate was 13.1% and the seroprevalence of HCV among prisoners with a history of injecting drug use (IDU) was 79.7%. Risk factors significantly associated with past HCV infection were IDU (p < 0.0001), having received a prison tattoo (p < 0.0001) or a non-sterile community tattoo (p < 0.0001), sharing needles and other drug-taking paraphernalia (p < 0.0001). Small numbers of prisoners had a history of sharing razors (n=10; 3.4%) and toothbrushes (n=3; 1.0%) while incarcerated. On multivariable analysis, history of receiving a non-sterile community tattoo was the only significant risk factor associated with HCV acquisition (after IDU was removed from the model) (p = 0.005, β = 0.468).

**Conclusion:**

The level of untreated chronic HCV infection in Irish prisons is high, with IDU the main associated risk.

## Introduction

Hepatitis C virus (HCV) infection is a major public health concern and a leading cause of liver-related morbidity and mortality globally [1]. There are an estimated 5.6 million people (1.1% of the population) living with chronic HCV infection in the World Health Organization European region [2]. In Europe, injecting drug use (IDU) is the major driver of this blood-borne infectious disease [3-5].

It is accepted that people who inject drugs (PWID) and HCV infection are typically over-represented in prison populations across Europe [6,7]. A recently published meta-analysis reported a prison HCV prevalence in Western Europe of 15.5% (95% confidence interval (CI): 12.2–19.1), with this prevalence increasing to over 40% among those prisoners with a history of IDU [8]. Although prisoners have been identified as a key at-risk group for HCV infection, epidemiological data on the extent of the HCV burden in prisons is incomplete, out of date and, in some European countries, non-existent [6,9]. Most epidemiological studies in people who inject drugs (PWID) and prisoners report on HCV antibody prevalence (exposure) and not presence of HCV RNA [6,10,11]. Where HCV RNA prevalence is reported, it is often on subsets of prisoners, and published studies do not differentiate between treated chronic infection with sustained viral response (SVR) and the 20–30% of HCV-infected people who spontaneously clear HCV without treatment [12].

Of the 600,000 people incarcerated in the European Union and European Economic Area (EU/EEA) at any given time, 3,400 are in prisons in Ireland [13]. A study in the year 2000 estimated the prevalence of HCV infection in the Irish prison population at 37%, increasing to 81% among those with a history of IDU [14]. A later study in 2014 reported an HCV prevalence of 13% [10]. This reducing trend in these two similar national studies is in keeping with a recognised global reduction in the prison prevalence of HCV and may in part be due to the expansion of community- and prison-based harm reduction services in Ireland [6,10].There is no systematic approach to HCV screening in Irish prisons and only three Irish prisons (including the study location) have specialist in-reach hepatology services. No acute HCV infection has been diagnosed at the study site in the past decade. Despite national and international guidelines recommending HCV screening for all prisoners and a community equivalence of care in relation to HCV treatment access, most Irish prisoners are not screened or treated for HCV infection [15-17].

This study aimed to estimate the levels of untreated chronic HCV infection and associated risk factors for acquisition in an Irish prison population. It updates previous prevalence studies and is unique both nationally and internationally in reporting genotype distribution and estimating levels of untreated chronic infection in a prison population.

## Methods

This study is reported in accordance with the Strengthening the Reporting of Observational studies in Epidemiology guidance (STROBE) [18]. Ethical approval for the research was granted by The Mater Misericordiae University Hospital Research Ethics Committee (Ref:1/378/1839) and the Irish Prison Service (IPS) Ethics Review Committee.

The Irish Prison Service (IPS) partnered with the European Commission Third Health Programme-funded HepCare Europe project [19] to enhance screening for populations at risk of HCV infection and specifically implement an enhanced HCV screening programme at Mountjoy Prison in Dublin, Ireland. Mountjoy Prison is a large urban prison which at capacity houses 538 sentenced male prisoners. The mean age of prisoners incarcerated at this location is 34 years, with a third serving sentences of less than 12 months and almost half on restricted regimes (protection prisoners) [13,20].

The programme consisted of an initial nurse-led screening programme followed by a peer-supported mass screening initiative. The Irish Red Cross trains a network of prison volunteers in basic community and public health across the IPS. Volunteers incarcerated at the study location collaborated in the design and implementation of this initiative which began in early 2017 and because of staffing and logistical issues required a 6-month period to complete.

All prisoners were offered screening for blood-borne viruses, including reflex RNA testing and genotyping. Reflex RNA testing is the automatic RNA testing of HCV antibody-positive samples without the need for a further request or repeat blood test.

### Laboratory tests

First-line serological screening for hepatitis B virus (HBV), HCV and human immunodeficiency virus (HIV) was carried out using chemiluminescent microparticle immunoassays on the Architect i4000sr automated platform (Abbott, Chicago, United States). Confirmatory testing of reactive samples was carried out using alternative assays. HIV serological diagnosis was based on the Architect HIV Ag/Ab Combo (Abbott, Chicago, United States) and confirmatory testing for reactive samples using the VIDAS HIV DUO Ultra (BioMérieux, Marcy-l'Étoile, France) and HIV INNO-LIA HIV I/II Score (Innogenetics, Ghent, Belgium ) assays. The Abbott Architect assays were used for HBV-associated markers and to characterise the HBV infection status. Serological screening for HCV included the anti-HCV test (Abbott Architect), a third-generation immunoassay and the Abbott Architect HCV Ag assay. All anti-HCV reactive samples negative for HCV antigen were further investigated to confirm the presence of anti-HCV antibodies using the anti-HCV VIDAS (BioMérieux) and INNO-LIA HCV Score (Innogenetics) assays. When HCV was confirmed serologically, molecular detection of HCV RNA was performed using the Abbott RealTi*me* HCV assay. HCV genotyping was conducted on samples with detectable HCV RNA using the Abbott RealTi*me* HCV Genotype II assay.

For this study, SVR was defined as HCV RNA-negative status at 24 weeks after completion of interferon-based treatment and 12 weeks for direct-acting antiviral (DAA) treatment. Chronic HCV infection was defined as ongoing active infection (HCV RNA-positive) 6 months or more after initial exposure (self-reported data from prisoners’ electronic medical records stored in the Prison Health Management System (PHMS)). A review of historical serology and HCV risk history stored in the PHMS was conducted for prisoners testing HCV antibody-positive. This allowed the HCV serology to be interpreted as infection with self-clearance, infection with SVR or untreated chronic infection. This information was cross-checked with the prisoner for accuracy.

### Data collection and analysis 

Data on variables were collected from two sources; prisoners’ electronic medical records stored in the PHMS and a researcher-administered questionnaire (Supplement 1). All prisoners routinely complete a nurse committal interview on the day of incarceration which is stored in the prisoners’ medical records in the PHMS. From this medical review, we collected the following variables: age, country of origin, history of drug and alcohol use, presence of visible injecting marks and history of sharing needles.

The questionnaire was developed and piloted by the research team in conjunction with national experts in the area of HCV infection and prisoner groups. The questionnaire included questions on age, country of origin, incarceration history, pre-incarceration accommodation, drug use, IDU history including history of sharing needles and drug-taking paraphernalia, history of tattooing, sharing of toothbrushes and razors while incarcerated and self-declared risk of HCV infection. Questionnaires were completed as part of screening but priority was given to screening, particularly in areas of the prison that housed enhanced-security prisoners.

All prisoners incarcerated on 31 March 2017 were eligible for inclusion in the study except those undergoing active treatment for severe mental illness and prisoners considered to pose a security risk to the research team. All study participants were given a patient information sheet and asked to sign a consent form. No inducements were offered.

All data were anonymised and coded, double-entered and checked. Statistical analysis was performed using the Statistical Package for Social Sciences (version 23.0; SPSS UK Ltd.; Chersey, United Kingdom). Data were assessed for normality and where necessary, data were log-transformed for normalisation purposes. Data in tables are primarily expressed as means with standard deviation (SD) or numbers with percentages. Categorical variables were assessed by chi-squared analysis (or Fisher’s exact test for n < 6) while nominal variables were assessed by independent t-test. Linear regression analysis adjusted for a history of IDU was used to identify possible risk factors for HCV infection (all variables initially selected, but then only the variables that resulted in the best model fit (R^2^) were left in the final model. 

## Results

### Demographic data

A total of 538 men were incarcerated on the day of the study, of whom 13 were excluded on mental health grounds, 58 refused consent to participate, 24 were released and 21 transferred to another prison before engagement in the study. A total of 422 (78.0% of the eligible population) participated in the study (Figure). Committal interview data was available for all 422 participants, 404 (95.7% of study participants) completed screening, and 298 (73.8% of those screened) completed some or the entire risk questionnaire (Figure).

**Figure:**
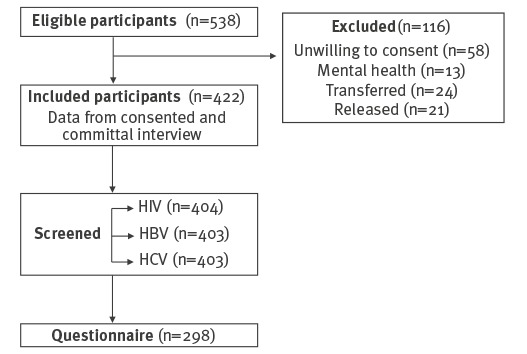
Study design for hepatitis C prevalence study, Mountjoy Prison, Ireland, 31 March 2017

HBV: hepatitis B virus; HCV: hepatitis C virus; HIV: human immunodeficiency virus.

The median age of the study population was 33.3 years (range: 18–63), 394 (93.4%) were Irish and the remainder came from numerous other countries. There were no statistical differences for age and country of origin between the entire prison population and the study recruits. At the nurse committal interview (n = 422), 208 (49.3%) admitted to a history of drug use, 27 (6.4%) had visible active injecting sites and 30 (7.1%) reported a history of sharing needles.

Data from the questionnaire (Table 1) showed a mean age at first incarceration of 19.6 years, with the mean number of incarcerations being 5.9 and a total mean time spent in prison of 8 years. The mean age at first drug use, heroin use and IDU was 15.3 years, 19.5 years and 20.5 years, respectively. Of those prisoners who completed questions on HCV risk factors, 134 (46.7%) indicated a history of heroin use and 90 (32.6%) a history of IDU, 44 (15.9%) admitted to sharing needles and 104 (37.4%) to sharing drug-taking paraphernalia. In addition, 48 reported getting a tattoo in prison (16.5%) and 46 a non-sterile tattoo in the community (16.0%). Only 10 prisoners (3.4%) reported sharing razors and three (1%) a toothbrush. Overall 96 prisoners (34.7%) reported ever engaging with methadone maintenance treatment (MMT), with the mean length of time on treatment being 6.2 years (Table 1).

**Table 1:** Demographic data of study participants, hepatitis C seroprevalence, Mountjoy Prison, Ireland, 31 March 2017–31 August 2017 (n = 422)

Variable	Participants			
	Total	n	%	Mean (SD)
Age (years)				
18–24	422	68	16.1	33.3 (8.7)
25–34		203	48.1	
≥ 35		151	35.8	
Age at first incarceration (years)	298			19.6 (6.8)
Episodes of incarceration	294			5.9 (6.6)
Total time incarcerated (years)	293			8.0 (6.8)
Age at first drug use (years)	233			15.3 (8.2)
Age at first heroin use (years)	202			19.5 (6.0)
Age at first injecting drug use (years)	86			20.5 (5.2)
Previous drug use^a^	422	208	49.3	
Visible injection site^a^	422	27	6.4	
Shared needles^a^	422	30	7.1	
Place of origin				
Ireland	422	394	93.4	
Western Europe		4	0.9	
Eastern Europe		17	4.0	
Africa		7	1.7	
Accommodation before incarceration				
Secure	258	210	81.4	
Homeless		48	18.6	
Risk factors for HCV acquisition				
History of heroin use	287	134	46.7	
History of injecting drug use	276	90	32.6	
Shared needle in the community	277	44	15.9	
Shared drug-taking equipment in the community	278	104	37.4	
Shared razor in prison	291	10	3.4	
Shared toothbrush in prison	292	3	1.0	
Prison tattoo	291	48	16.5	
Unsterile community tattoo	287	46	16.0	
Alcohol use				
Alcohol problem before incarceration	293	50	17.1	
Treatment for alcohol use	196	18	9.2	
Methadone maintenance treatment				
History of methadone treatment	277	96	34.7	
Length of time on methadone maintenance treatment	96			6.2 (5.8)

HCV: hepatitis C virus; SD: standard deviation.

Values are means (± SD) for continuous variables or n (%) for categorical variables.

^a^ Data from committal interview.

#### Blood-borne virus status

Of the 422 study participants, 404 (95.7%) had a screen for HIV and 403 (95.5%) each for HBV and HCV infection, with respective prevalences of 4.0%, 3.0% and 23.8%. Of the 92 HCV antibody-positive prisoners, 10 (11%) were co-infected with HIV and 6 (6.5%) with HBV. Prisoners with a history of IDU had an HCV antibody prevalence of 79.7%.

Among the HCV antibody-positive prisoners, 23 (25.0%) had spontaneous clearance, 16 (17.4%) had an SVR after treatment and the remaining 53 (57.6) had untreated chronic infection. This reflects an untreated chronic HCV infection prevalence of 13.2% among the 403 tested participants (Table 2).

**Table 2:** Blood-borne virus screening results of study participants, Mountjoy Prison, Ireland, 31 March 2017–31 August 2017 (n = 404)

Variable	Participants		
	Total	n	%
HIV antibody			
Positive	404	16	4.0
Negative		388	96.0
HBV antibody			
Positive	403	12	3.0
Negative		391	97.0
HCV antibody			
Positive	403	92	22.8
Negative		311	77.2
Reflex RNA testing			
Positive (untreated chronic infection)	92	53	57.6
Negative		39	42.4
SVR post treatment		23	25.0
Spontaneous clearance		16	17.4
Genotype			
1a	63	37	58.7
3		26	41.3

HBV: hepatitis B virus; HCV: hepatitis C virus; HIV: human immunodeficiency virus; SVR: sustained viral response.

Genotyping was available for 63 (50 from screening and 13 from medical records) of those with chronic infection (treated and untreated); 37 were infected with Genotype 1A and 26 with Genotype 3 (Table 1).

#### Associations for hepatitis C virus infection

On comparative analysis (combined from committal interview and questionnaire) there were significant differences between those who were HCV antibody-positive and those who were not (Table 3). HCV antibody-positive prisoners were older (p < 0.0001), had more episodes of incarceration (p < 0.0001), had spent longer in prison (p < 0.0001) and were younger at first heroin use (p = 0.0003) and IDU (p = 0.015). Stability of accommodation before incarceration was a factor for HCV status, with those experiencing homelessness more likely to be infected (p = 0.001). Country of origin did not impact on HCV status, with no statistical differences seen between Irish and non-Irish prisoners (p = 0.593). We could not examine the association with shared razors or toothbrushes in prison because of the small numbers.

**Table 3:** Predictors of hepatitis C virus antibody status in study participants, Mountjoy Prison, Ireland, 31 March 2017–31 August 2017 (n = 403)

Category	Variable	Positive		Negative		p value
		n	%	n	%	
Age in years (SD)		39.3 (6.9)		31.4 (8.4)		<0.0001
Place of origin (C)	Ireland	85	92.3	290	93.2	0.593
	Western Europe	1	1.1	3	1	
	Eastern Europe	3	3.3	14	4.5	
	Africa	3	3.3	4	1.3	
Incarceration history (C)	Age at first incarceration, in years (SD)	18.2 (6.5)		20(6.9)		0.067
	Episodes of incarceration	9.6	9.1	4.8	5.5	<0.0001
	Total time incarcerated, in years (SD)	12.6 (7.8)		6.5 (5.6)		<0.0001
Accommodation before incarceration (Q)	Secure	35	66	166	85.6	0.001
	Homeless	18	34	28	14.4	
Drug history (C)	Previous drug use (yes)	78	85.7	122	39.2	<0.0001
	Visible injection site (yes)	16	17.6	10	3.2	<0.0001
	Shared needles (yes)	23	25.3	6	1.9	<0.0001
	History of alcohol use (yes)	16	17.6	29	9.3	0.028
Drug history (Q)	Age at first drug use in years (SD)	15.1 (4.4)		14.9 (4.9)		0.733
	Age at first heroin use in years (SD)	17.7 (4.5)		20.7 (6.7)		0.003
	Age at first injecting drug use in years (SD)	19.3 (4.9)		22.1 (5.2)		0.015
	Time on MMT in years (SD)	8 (8.0)		3.5 (4.4)		0.005
	History of drug use (yes)	58	96.7	175	79.2	0.001
	History of heroin use (yes)	52	91.2	76	35.3	<0.0001
	History of injecting drug use (yes)	47	79.7	41	20.3	<0.0001
	Alcohol problem before incarceration (yes)	13	22	35	16	0.275
	Treatment for alcohol use (yes)	4	11.4	14	9.1	0.671
	MMT history	45	81.8	47	22.7	<0.0001
Reported HCV risk (Q)	Shared needle in community	32	55.2	11	5.4	<0.0001
	Shared drug-taking equipment in community	42	73.7	54	26.2	<0.0001
	Prison tattoo	19	32.8	27	12.4	<0.0001
	Unsterile tattoo in community	15	25.9	29	13.6	0.024
	Shared razor in prison	2	3.4	7	3.2	N/A
	Shared toothbrush in prison	1	1.7	1	0.5	N/A
Blood-borne virus results	HIV antibody result					
	Positive	10	11	6	1.9	<0.0001
	Negative	81	89	303	98.1	
	HBV antibody result					
	Positive	6	6.6	6	1.9	0.023
	Negative	85	93.4	302	98.1	

C: committal interview; HBV: hepatitis B virus; HCV: hepatitis C virus; HIV: human immunodeficiency virus; MMT: methadone maintenance treatment; N/A: not applicable; Q: questionnaire; SD: standard deviation.

Values are means (±SD) for continuous variables or n (%) for categorical variables. Denominators for each variable represent participants for whom data was available. Student’s independent t test was used to test differences between continuous variables. Chi-square tests were used to test differences between categorical variables.

HCV antibody-positive prisoners were significantly more likely to have a history of drug use (p = 0.001) or of heroin use, IDU or sharing needles or drug-taking paraphernalia in the community (p < 0.0001). HCV antibody-positive prisoners were more likely to have had a prison tattoo (p < 0.0001) and an unsterile community tattoo (p = 0.024). A pre-incarceration history of, and treatment for, alcohol misuse problems did not impact on prisoners’ HCV status (p = 0.275 and p = 0.671, respectively). HCV antibody-positive prisoners were also significantly more likely to have a history of MMT (p < 0.0001).

On preliminary multivariable analysis (including IDU), none of the above risk factors were independent risk factors for HCV infection (Supplement 2). When IDU was excluded, only getting a non-sterile tattoo in the community remained an independent risk factor (p = 0.005) (Table 4).

**Table 4:** Regression model, hepatitis C study, Mountjoy Prison, Ireland, 31 March 2017–31 August 2017 (n = 403 with all variables of interest)

	B	SE B	β	*t*	p value
Constant	1.188	0.33	N/A	3.599	0.001
Shared drug-taking equipment in the community	0.023	0.097	0.035	0.231	0.818
Prison tattoo	−0.047	0.139	−0.052	−0.34	0.736
Unsterile community tattoo	0.468	0.157	0.468	2.977	0.005
Homelessness	−0.075	0.082	−0.13	−0.922	0.362

B: beta; β: standardised version of B; HCV: hepatitis C virus; N/A: not applicable; SE B: standard error of B; t: t-statistic dependent variable: HCV antibody result.

## Discussion

This study found an HCV antibody prevalence of 22.8% in the general prison population of an Irish male prison, increasing to 79% among prisoners with a history of IDU. A quarter of the HCV-exposed cohort had spontaneously cleared the virus and 17.4% had an SVR post-treatment. Reflex RNA testing showed that 57.3% of HCV exposed prisoners had untreated chronic infection, which represents a prevalence of 13.1% untreated chronic HCV infection in the general prison population. The main risk for HCV acquisition was IDU (p < 0.0001). This study found low levels of self-reported sharing of tooth brushes and razors. 

The HCV antibody prevalence of 22.8% in this study is comparable to that reported in a review estimating the prevalence among prisoners globally [6]. It is higher than the 13% reported in the last Irish prison-based HCV prevalence study in 2014 [10] but lower than the 37% reported in an earlier study in 2000 [14]. Both these studies were multi-site national studies which included most of the 15 prisons in Ireland. The downward trend after the study in 2000 [14] is supported by the findings of the global 2013 systematic review [6] and a more recent European review which found that older studies reported higher prevalence rates [3].

The upward trend since the 2014 study may be explained by the fact that ours was a single-site study, in a prison considered to have moderate to high levels of PWID among its prison population and that consequently would be expected to have a higher HCV disease burden [6,10,14]. This finding also underpins the inter-prison and regional variation in HCV prevalence that occurs within and between countries and the need for accurate and institution-specific data when planning and resourcing prison-based HCV treatment services [6,10,14]. The HCV prevalence we estimated here reflects a rate 50 times higher than the estimated HCV prevalence in the general Irish population [21].

There is marked regional variation and extensive gaps in the data on the active HCV disease burden in prisoners [6]. Most prison-based prevalence studies report on HCV exposure rather than active infection [6,10]. Of those exposed, 20–30% will spontaneously clear the virus and the remainder will progress to chronic infection, which is measured by the presence of HCV RNA in the blood 6 months after the acute infection [22]. Our study found a spontaneous clearance of 25%, similar to previously published studies [1,22,23].

We found that 23.2% of those with chronic infection had been successfully treated with SVR and not had re-infections to date. This level of treatment is high compared with other reported studies and may reflect the impact of an in-reach hepatology service at this site for many years [24,25]. This finding also supports the existing evidence that PWID and prisoners can be successfully treated for HCV infection [26,27]. The absence of re-infection among those showing SVR after treatment is contrary to published evidence showing high levels of re-infection among prisoners [28]. This may in part be due to the high levels of methadone maintenance treatment in Irish prisons. A study in Scotland reported that incarceration was protective against HCV transmission because of the high levels of opioid substitution treatment coverage in Scottish prisons [29].

In the era of interferon-based HCV treatment, so few PWID and prisoners were treated that HCV antibody prevalence data, mathematically modelled to allow for spontaneous clearance, reflected the untreated chronic HCV disease burden [6]. With the new DAA, therapies many countries are engaging significant numbers of prisoners and PWID in treatment with high compliance and SVR rates [30-32] Studies that report on HCV antibody levels in populations do not distinguish between self-clearance and SVR post treatment [12,33] and miss the impact of treatment and rates of re-infection [12,33]. This unique study demonstrates how more detailed HCV surveillance data can more accurately estimate the HCV disease burden in a population.

As previously outlined, drug users and PWID are over-represented in prison populations globally [6,10]). This study reports similar findings with half of prisoners admitting to drug use and a third to a history of IDU. In this prison cohort, drug use began at an early age, with progression to heroin use and IDU within 5 years. High levels of risk behaviour related to drug use were also reported, including sharing needles and other drug-taking paraphernalia. These findings reflect the reality of drug use and risk behaviour in this marginalised cohort and indicate that prevention strategies are needed from a very young age.

A number of HCV transmission risks in prisoners have already been reported in the literature; these include IDU, prison tattooing and factors independent of these but linked to incarceration, such as violent assault, sharing of toothbrushes and razors [6,10,34,35]. Our study reports IDU as being the main risk factor for HCV infection. A number of other variables (age, homelessness, length of time on methadone, length of time and numbers of times incarcerated, sharing of non-needle drug-taking paraphernalia and prison tattooing) were statistically associated with HCV exposure but in multivariable regression analysis not independent of IDU. This suggests that prisoners with a history of IDU have multiple HCV risks and that any planned HCV prevention strategies will need to target all these factors [6,34,36,37]. 

Targeting prisoners with a history of IDU for active HCV case finding is a priority given the high HCV prevalence rates in this group [7]. It is also important to recognise that prisoners will under-report IDU because of fear of stigma and discrimination [6,10,12]. The move away from risk-based to opt-out prison-based HCV screening is addressing this issue in some jurisdictions and has the potential to be adapted by the IPS to increase screening uptake among Irish prisoners [38,39].

Being older has previously been reported as a risk factor for past HCV infection [6,10,35]. We report similar findings for age but found that this was not independent of IDU. Again, this is expected given that older prisoners have longer histories of IDU with an elevated risk of potential HCV exposure over time. The finding that the vast majority of prisoners do not share toothbrushes or razors is encouraging and certainly in this particular prison eliminates these as sources of HCV transmission. Previous studies have reported high HCV prevalence among prisoners coming from countries of high HCV endemicity [6,34,40]. This study shows that being non-Irish in this prison population was not associated with HCV exposure. However, the numbers for this were small and had to be categorised for reporting in a way that protected anonymity. 

The 78% participation is a major strength of this study. Previous Irish HCV prevalence studies found the prisoners in this particular prison difficult to engage and had much poorer uptake [10]. The use of prison-based peer workers in this study may account for the improved uptake. These studies were randomised in design and did not include the entire population. The use of serum samples rather than historical blood-borne virus screening data from chart records is unusual in these types of studies and with high levels of both sensitivity and specificity for this method offers increased validity to this study over those using saliva samples [10,14].

The use of a research-completed questionnaire has both strengths and limitations. The researchers completing the questionnaire were unknown to the prisoners which may have allowed for more frank disclosure of risk behaviour. The two previous Irish studies used self-completed questionnaires. The design of our study allowed researchers to spend time with the prisoner and to add clarification regarding the meaning/interpretation of the questions. While overcoming the issue of literacy, it was time-consuming which impacted on completion rates.

There are a number of limitations to this study. It was single-site and only included male prisoners, making the findings more difficult to generalise both nationally and internationally. The study was designed to allow for its implementation in a real prison setting and for it to be both a health intervention (HCV screening) and a research study measuring HCV prevalence. The dynamic nature of prison populations (releases, inter-prison transfers and short sentences) makes cross-sectional epidemiological studies difficult to implement. In addition, owing to the nature of the study we did have a small ‘n’ for a number of variables of interest. Where statistically possible, we tried to keep important variables in statistical models and we did utilise tests for normality for small sizes and test model quality. However caution must be used in the interpretation of the results until they are replicated in larger studies.

The self-reported nature of the data is a limitation. We did not receive ethical approval to retrieve data on study participants’ incarceration history from their electronic prison records and therefore had to rely on self-reporting for these variables which may be prone to bias. There were limits on allocated time to access prisoners; consequently, screening was prioritised over the completion of the risk questionnaire. This was particularly an issue with protection prisoners where enhanced security measures were in place.

## Conclusion

This study reports an anti-HCV prevalence of 22.8%, with an untreated chronic HCV prevalence of 13.2% in a male Irish prison population. The identified genotype distribution was 58% genotype 1A and 42% genotype 3A. The main risk for HCV infection was IDU but there was also a statistically significant association with prison tattooing, non-sterile community tattooing, the sharing of needles and other drug-taking paraphernalia, older age and pre-incarceration homelessness. The sharing of toothbrushes and razors or not having Ireland as a country of origin were not associated with HCV exposure. The reporting of HCV infection in prisoners in this way is unique in both the Irish and international literature and allows for the estimation of the true levels of active HCV infection, the monitoring of treatment outcomes and rates of re-infection. Identifying risk factors for HCV infection allows for targeted prevention, screening and treatment strategies. Combined, they allow informed planning and implementation of national and international HCV strategies.

## Supplementary Data

453000 Supplement1

## Supplementary Data

91000 Supplement2
