# Supplementary material for: The seroprevalence of untreated chronic hepatitis C virus (HCV) infection and associated risk factors in male Irish prisoners: a cross-sectional study, 2017
Source: Euro Surveill. 2019 Apr 4;24(14):1800369. doi: 10.2807/1560-7917.ES.2019.24.14.1800369 (PMC6462789; doi:10.2807/1560-7917.ES.2019.24.14.1800369)
Supplement: Supplement1 [file 18-00369_CROWLEY_Supplement1.pdf]

IRISH PRISON SERVICE

Supplement 1: Questionnaire

This supplementary material is hosted by Eurosurveillance as supporting information alongside the article 'The seroprevalence of chronic untreated hepatitis C virus (HCV) infection and associated risk factors in Irish prisoners: a cross-sectional study'on behalf of the authors who remain responsible for the accuracy and appropriateness of the content. The same standards for ethics, copyright, attributions and permissions as for the article apply. Eurosurveillance is not responsible for the maintenance of any links or email addresses provided therein."

Prison HCV Risk Questionnaire

Part 1

1(a) Initials	
1 (b) D.O.B	
1(c) PRIS Number	
1(d)Nationality	
1 (e) Age at First Incarceration	
1 (f) Number of episodes of Incarceration	
1 (g) Length of time spent in Incarceration	

Part 2 - HCV Risk

Please circle your answer

2(a) Do you consider yourself at risk of HCV?<br>If no go directly to question.	**Yes** **No**
2 (b) If yes, what are your risk factors?	IV Drug Use ☐<br>Sharing needles/drug taking paraphernalia ☐<br>Non-Sterile tattooing ☐<br>Receiving infected blood products ☐<br>Had sex with a man (MSM) ☐<br><br>Other ____________

Part 3 – Drug History and Risks

Supplement 1: Questionnaire

This supplementary material is hosted by Eurosurveillance as supporting information alongside the article 'The seroprevalence of chronic untreated hepatitis C virus (HCV) infection and associated risk factors in Irish prisoners: a cross-sectional study'on behalf of the authors who remain responsible for the accuracy and appropriateness of the content. The same standards for ethics, copyright, attributions and permissions as for the article apply. Eurosurveillance is not responsible for the maintenance of any links or email addresses provided therein."

3 (a) Do you have a history of drug use?	**Yes** **No**
3(b) If yes, age of first drug use	
3 (c) Do you have a history of heroin use?	**Yes** **No**
3 (d) If yes, age of first heroin use	
3 (e) Do you have a history of IV use?	**Yes** **No**
3 (f)If yes, age of first IV use	
3 (g) Do you have a history of needle/syringe sharing in the community?	**Yes** **No**
3 (h) History of sharing drug taking paraphernalia in the community?	**Yes** **No**
3 (i) First IV Use in prison?	**Yes** **No**
3(j) IV use in prison?	**Yes** **No**
3(k) If yes- how long ago?	
3 (l) Any IV steroid use in prison?	**Yes** **No**
3 (m) Do you have a history of needle/syringe sharing in prison?	**Yes** **No**
3 (n) History of sharing drug taking paraphernalia in the prison?	**Yes** **No**
3 (o) Have you ever shared a razor or tooth brush while in prison?	**Yes** **No**
3 (p) History of tattooing in prison?	**Yes** **No**

Part 4 – Drug Treatment

IRISH PRISON SERVICE

Supplement 1: Questionnaire

This supplementary material is hosted by Eurosurveillance as supporting information alongside the article 'The seroprevalence of chronic untreated hepatitis C virus (HCV) infection and associated risk factors in Irish prisoners: a cross-sectional study'on behalf of the authors who remain responsible for the accuracy and appropriateness of the content. The same standards for ethics, copyright, attributions and permissions as for the article apply. Eurosurveillance is not responsible for the maintenance of any links or email addresses provided therein."

4 (a) Are you on MMT?	**Yes** **No**
4 (b) If yes, total length of time on MMT	
4(c) If no, MMT in the past?	**Yes** **No**

Part 5 – HCV status

5(a) Have you had a HCV blood test?	**Yes** **No**

5(b) Where did you get the test?	
5 (c) How long ago was it?	
5 (d) Have you had a risk of Infection since test?	**Yes** **No**
5 (e) What was the result?	**Pos** **Neg** **Not sure/UK**
5(f) Are you aware of your antigen/PCR status?<br>If yes	**Yes** **No**
5 (g) Result of antigen /PCR	**Pos** **Neg**
5 (h) Were you referred for treatment?	**Yes** **No**
5 (i) Did you attend treatment?	**Yes** **No**
5(j) If no, why not?<br>Record no more than 5 reasons	**1** ____________<br>**2**____________<br>**3**____________<br>**4**____________<br>**5**____________

IRISH PRISON SERVICE

Supplement 1: Questionnaire

This supplementary material is hosted by Eurosurveillance as supporting information alongside the article 'The seroprevalence of chronic untreated hepatitis C virus (HCV) infection and associated risk factors in Irish prisoners: a cross-sectional study'on behalf of the authors who remain responsible for the accuracy and appropriateness of the content. The same standards for ethics, copyright, attributions and permissions as for the article apply. Eurosurveillance is not responsible for the maintenance of any links or email addresses provided therein."

Part 6

6(a) Would you like to have a HCV test?	**Yes**	**No**
6 (b) If yes,	**Oral Swab**	**Blood test**
6(c) If bloods previously taken, would you give written consent for us to access bloods from the VRL.<br>If yes – please sign release of information form	**Yes**	**No**

Signed ________________________ Date:
