# Supplementary material for: The seroprevalence of untreated chronic hepatitis C virus (HCV) infection and associated risk factors in male Irish prisoners: a cross-sectional study, 2017
Source: Euro Surveill. 2019 Apr 4;24(14):1800369. doi: 10.2807/1560-7917.ES.2019.24.14.1800369 (PMC6462789; doi:10.2807/1560-7917.ES.2019.24.14.1800369)
Supplement: Supplement2 [file 18-00369_CROWLEY_Supplement2.pdf]

Supp. 2: Regression Model investigating independent risk factors for HCV infection*

This supplementary material is hosted by Eurosurveillance as supporting information alongside the article 'The seroprevalence of chronic untreated hepatitis C virus (HCV) infection and associated risk factors in Irish prisoners: a cross-sectional study'on behalf of the authors who remain responsible for the accuracy and appropriateness of the content. The same standards for ethics, copyright, attributions and permissions as for the article apply. Eurosurveillance is not responsible for the maintenance of any links or email addresses provided therein."

	**Beta**	**P value**
Total time	-0.115	0.071
History drug use	-61.411	0.999
Age 1st drug use	0.079	0.464
History IV use	-21.53	0.998
Shared needles	-39.355	0.998
Shared equipment	-1.09	0.262
Shared toothbrush	-1.848	1
Methadone treatment history	0.229	1
Time on MMT	0.002	0.969

*Independent of Hx of IDU
